# A Novel Bismuth-Chitosan Nanocomposite Sensor for Simultaneous Detection of Pb(II), Cd(II) and Zn(II) in Wastewater

**DOI:** 10.3390/mi10080511

**Published:** 2019-07-31

**Authors:** Jae-Hoon Hwang, Pawan Pathak, Xiaochen Wang, Kelsey L. Rodriguez, Hyoung J. Cho, Woo Hyoung Lee

**Affiliations:** 1 Department of Civil, Environmental, and Construction Engineering, University of Central Florida, Orlando, FL 32816, USA; 2 Department of Mechanical and Aerospace Engineering, University of Central Florida, Orlando, FL 32816, USA; 3 Department of Materials Science and Engineering, University of Central Florida, Orlando, FL 32816, USA

**Keywords:** nanocomposite films, environmental sensors, co-electrodeposition, heavy metal ions, mining wastewater, soil leachates

## Abstract

A novel bismuth (Bi)-biopolymer (chitosan) nanocomposite screen-printed carbon electrode was developed using a Bi and chitosan co-electrodepositing technique for detecting multiple heavy metal ions. The developed sensor was fabricated with environmentally benign materials and processes. In real wastewater, heavy metal detection was evaluated by the developed sensor using square wave anodic stripping voltammetry (SWASV). The nanocomposite sensor showed the detection limit of 0.1 ppb Zn^2+^, 0.1 ppb Cd^2+^ and 0.2 ppb Pb^2+^ in stock solutions. The improved sensitivity of the Bi-chitosan nanocomposite sensor over previously reported Bi nanocomposite sensors was attributed to the role of chitosan. When used for real wastewater samples collected from a mining site and soil leachate, similar detection limit values with 0.4 ppb Cd^2+^ and 0.3 ppb Pb^2+^ were obtained with relative standard deviations (RSD) ranging from 1.3% to 5.6% (*n* = 8). Temperature changes (4 and 23 °C) showed no significant impact on sensor performance. Although Zn^2+^ in stock solutions was well measured by the sensor, the interference observed while detecting Zn^2+^ in the presence of Cu^2+^ was possibly due to the presence of Cu-Zn intermetallic species in mining wastewater. Overall, the developed sensor has the capability of monitoring multiple heavy metals in contaminated water samples without the need for complicated sample preparation or transportation of samples to a laboratory.

## 1. Introduction

Heavy metal ions such as lead, cadmium, zinc, and copper are well-studied water and soil pollutants that pose a threat to public health and have a lingering impact on various ecosystems. They are non-biodegradable and have long biological half-lives, thus lasting longer in aquatic and terrestrial environments, negatively affecting both humans and other living organisms. Currently, contamination by heavy metal ions has attracted increasing public awareness due to galvanic corrosion which contributes to raising lead levels in drinking water distribution systems [1]. With an increased concern over heavy metal contamination as a public health hazard, the regulations toward heavy metals in drinking water are becoming stricter (e.g., 0.015 ppm of Pb^2+^ and 1.3 ppm of Cu^2+^) [2]. Heavy metal contaminations can be found in liquid wastes of many related industries (e.g., mining operations and metal processing) [3]. The traditional methods such as electrothermal atomic absorption spectrometry (EAAS), flame atomic absorption spectrometry (FAAS), and inductively coupled plasma mass spectrometry (ICP-MS) and inductively coupled plasma (ICP)) have provided accuracy and reliability in detecting heavy metals. However, they often require large, expensive instruments, highly trained technicians, considerable time-consuming efforts, large volumes of reagents, an invasive collection of samples in the field, and transportation to centralized laboratories for analyses. The long-time delays associated with current procedures are considered inadequate for on-site applications and cannot be used for taking preventive measures in the early stages of the escalation of water crises. It is of vital importance to develop a quick, simple, reliable method for heavy metal detection that can be used for real-world applications [4]. 

Electrochemical detection is a unique and promising advancement in that it is suited for simple and rapid analysis and an ideal design for fabrication on small circuits in the form of portable devices [5,6]. Specifically, square wave anodic stripping voltammetry (SWASV) has been extensively studied for heavy metal detection in water [7,8]. Mercury (Hg) [9,10,11] and bismuth (Bi) [12,13,14,15] have been used as sensing materials due to their formation of amalgam with different analytes. Among them, Bi provides several advantages of partial insensitivity to dissolved oxygen (DO), and its ability to operate in high pH solutions, and the better separation between peaks in stripping analyses [16] over Hg which is not environmentally benign due to its high level of toxicity [17]. It is well studied that bismuth forms an alloy with transition metal ions instead of competing for the surface site [15]. The formation of the alloy is responsible for the excellent stripping performance of the bismuth-coated electrode [15]. However, its mechanical characteristics limit its functions as a robust electrode material due to its brittleness that often results in detachment of Bi from the base electrode [18]. 

Recently, the biopolymer (e.g., chitosan) has been recognized as a promising supplementation to Bi and other metal film electrodes due to its relatively high mechanical strength, good adhesion to traditional electrochemical surfaces, high permeability towards water, water solubility (at low pH), biocompatibility and low cost [19,20,21]. Additional advantageous properties include the formation of stable chelates with many transition metal ions due to the presence of hydroxyl and amino groups, which provide enhanced affinity to metal ions and improved detection sensitivity [22,23,24,25]. However, only a few reports on the use of Bi-chitosan composite are available, for example, in determining Sunset Yellow or Carmoisine in food colorants [26] and for DNA immobilization and hybridization detection [27]. The use of a Bi-chitosan composite film for heavy metal detection is still at an early developmental stage. Furthermore, in order to validate the environmental sensing applications of Bi-chistosan composite-based electrodes, a series of experiments are required to evaluate how well the sensors work in real contaminated water environments beyond preformulated stock solutions. In real deployment conditions, temperature is another parameter that is to be accounted for the analytical performance of a sensor, since it may affect the affinity and mobility of the metal ions in solution [28]; however, there are no reports on temperature effects of a Bi-chitosan composite sensor. The acidic drainage temperature reported is typically less than 5 °C for 3–5 months and 18–23 °C for 5–8 months of the year [29]. In this study, we developed a nanocomposite film with Bi and chitosan to increase both mechanical stability and electrochemical sensitivity for improved heavy metal detection. The developed sensor was then characterized for detecting Cd^2+^, Pb^2+^, and Zn^2+^ ions. The sensor performance was evaluated comprehensively in terms of representativeness, repeatability, limit of detection (LOD), lifetime and temperature effect with mining wastewater and contaminated soil leachate. 

## 2. Materials and Methods

### 2.1. Chemicals 

Carbon, silver, and insulating polymer pastes used for fabricating a screen printed sensor were purchased from Daejoo Electronic Materials Co. Ltd. (Gyeonggi-do, Korea). A 0.1 M acetate buffer solution (AcB) with pH 4.5 was prepared by mixing the premeditated amount of acetic acid in DI water. Stock solutions of 0.1 M Zn^2+^, Cd^2+^ and Pb^2+^ were prepared by dissolving the appropriate amount of cadmium (II) nitrate, lead (II) nitrate, and zinc (II) chloride in DI water. All other chemicals in this study were analytical grade, purchased from Sigma-Aldrich and used without further purification.

### 2.2. Bi-Chitosan Sensor Fabrication

**Figure 1:**
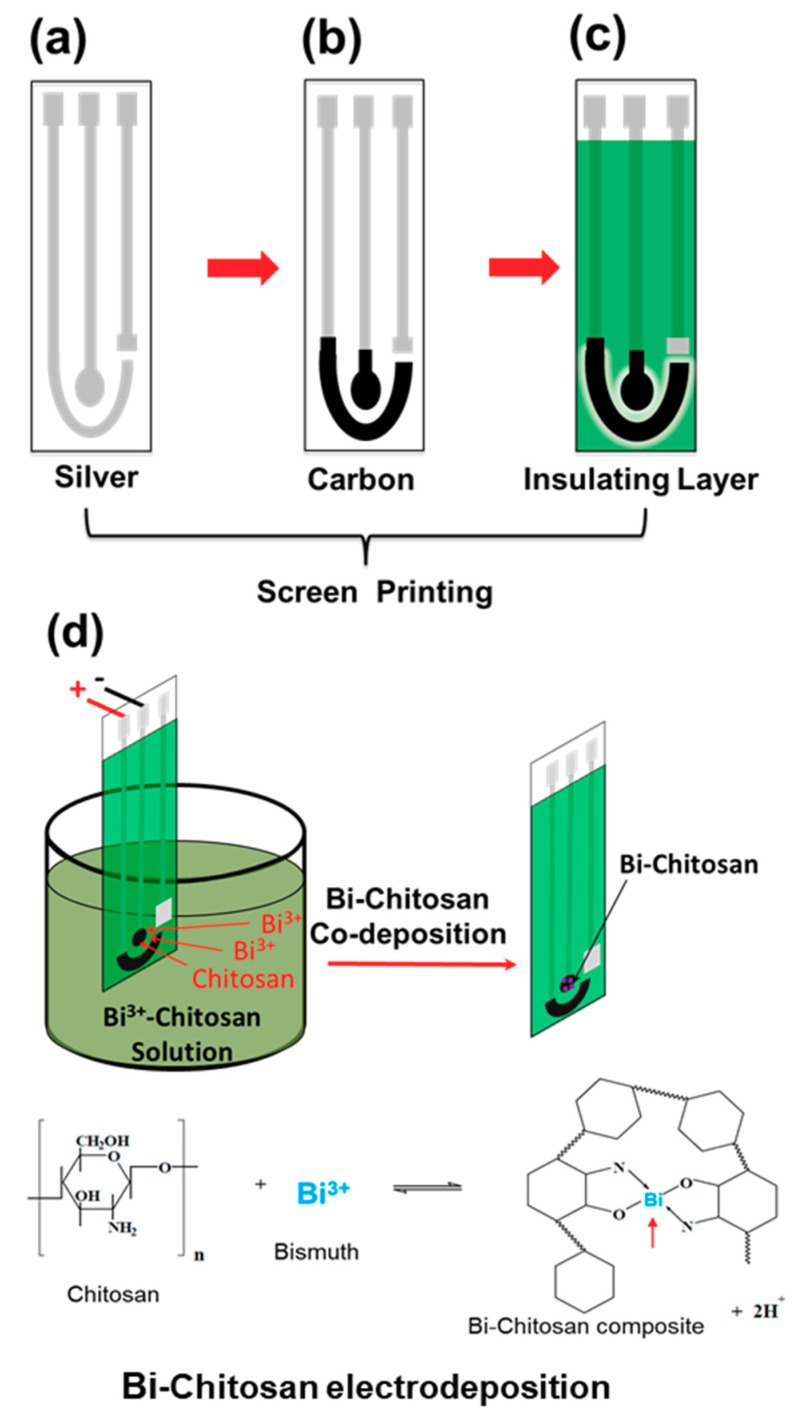
A schematic of the Bi-chitosan sensor fabrication process. (**a**) Silver layer, (**b**) carbon layer, (**c**) insulating layer fabricated by screen printing, and (**d**) electrochemical co-deposition of Bi-chitosan nanocomposite film.

For a low-cost substrate, a polyvinyl chloride (PVC) film (Grafix, Maple Heights, OH, USA) was used for screen printing of silver, carbon, and insulation layers. Figure 1 shows the screen-printing procedure and electrochemical co-deposition of Bi and chitosan on the working electrode (carbon). Three metal mesh screens for each layer formation were designed using AutoCAD and purchased from NBC Meshtec Americas Inc. (Batavia, IL, USA). For a screen-printing of three layers, a silver paste as a conductive layer was printed first on the PVC substrate and dried for 4 h at 23 °C. Next, a carbon paste was printed for forming two separate layers of the counter and the working electrode and were also dried for 4 h at 23 °C. Lastly, an insulating layer was formed to seal the silver conductive layer, excluding the working and counter electrodes. After curing the insulating layer with UV light (36 W) for 2 h, the screen printed electrodes were then polished using a DI water infused with 1 µm alumina particles to remove particle debris and provide a flat, isotropic surface [30,31]. Prior to electrodeposition, the polished electrodes were cleaned with DI water and desiccated at room temperature for 12 h.

Bi and chitosan were electrochemically co-deposited on the finished screen-printed carbon working electrode using a three-electrode configuration. An Ag/AgCl electrode (MI-401, Microelectrodes, Inc, Bedford, NH, USA) was used as an external reference electrode. The electrolyte used for co-deposition consisted of 24 mg of chitosan and 0.1M Bi nitrate in 20 mL of acetic acid solution with a concentration of 0.1 M (pH 4.5). The optimized weight ratio of bismuth with respect to chitosan in the electrolyte solution was ~16:1. The solution was continuously stirred for a 24-h period to ensure a well-mixed condition. Then, electrochemical co-deposition was performed under −100 mA/cm^2^ DC current supply for 30 to 180 s and at a current of −200 mA/cm^2^ DC for 120 s to produce a composite film. Finally, the composite film-coated sensor was rinsed with DI water, dried, and stored under ambient conditions before use. 

### 2.3. Characterization of Co-Deposited Bi-Chitosan Nanostructure

The optical images for Bi-chitosan films were obtained using a microscope (Balplan, Bausch & Lomb microscope, New York, NY, USA). The surface morphology of the composite and characterization of the films were observed using field emission scanning electron microscopy (FESEM) (Hitachi S4700 SEM, Hitachi America, Ltd., New York, NY, USA) and X-ray photoelectron spectroscopy (XPS), respectively. FESEM operated at an accelerating voltage of 10 kV and XPS was performed using an XR-50 dual anode Al/Mg K-alpha X-ray source with a Phoibos100 Hemispherical Analyzer (SPECS GmbH, Berlin, Germany). Monochromatic Al Kα line was used to analyze XPS of the sample. The chamber pressure was maintained below 1.9 × 10^−9^ torr during XPS analysis. Charge transfer resistance at electrode/electrolyte interface was studied using electrochemical impedance spectroscopy (EIS) with PalmSens 3 EIS (PalmSens Compact Electrochemical Interfaces, Houten, Netherlands). EIS measurements were performed using the three-electrode system under polarization potential of −400 mV vs. Ag/AgCl and frequency range 10 mHz to 500 kHz. The aqueous 10 mM potassium ferricyanide (K_3_[Fe(CN)_6_]) solution was used as the supporting electrolyte.

### 2.4. Electrochemical Heavy Metal Detection using SWASV

For sensor performance evaluation for heavy metal detection, a commercial electrochemical cell (10 mL, Compact Voltammetry Cell-Starter Kit, Pine instrument, Grove City, PA, USA) was used in a three electrode system (Bi-chitosan co-deposited surface as a working electrode, carbon counter electrode, and an Ag/AgCl electrode). A PalmSens 3 EIS was used as a potentiostat for sensor characterization and performance evaluation. Preliminary evaluation was conducted for scanning the working ranges of potential, deposition time, amplitude, and frequency for SWASV heavy metal detection using the developed sensor. For the detection of multiple heavy metals, Cd^2+^, Pb^2+^, and Zn^2+^ were first deposited at −1.2 V vs. Ag/AgCl simultaneously for 300 s in 0.1 M AcB (pH 4.5) (unless otherwise specified). Next, the deposited metals on the Bi-chitosan nanostructure film were stripped off under the optimized SWASV condition (i.e., a step potential of 4 mV, 50 mV amplitude, and a frequency of 20 Hz). For sequential measurements, the working electrode was cleaned at −0.3 V for 300 s before the next measurement to remove remnants of heavy metal ions that may have been deposited on the electrode surface in the previous measurement. The cleaning step showed no structural damage of the preformed Bi nanostructure on the working electrode surface. All experiments were performed in duplicate and data were expressed in terms of the mean ± standard deviation (SD). 

Limit of detection (LOD) was determined using the following equation: C_L_ = kS_B_/b (Equation (1)) [32], where C_L_ signifies the detection limit, S_B_ represents the standard deviation of blank signals, k is a parameter with a value of 3, and b is the slope of the calibration curves. 

### 2.5. Real Wastewater Samples

For the sensor evaluation in real environmental samples two wastewater samples were obtained: one from a mining site (Il-gwang mine) and the other from a soil contaminated site (Jukcheong mine) in South Korea [33]. Regarding soil leachate preparation, the soil from the Jukcheong mine site was first sieved using a #10 (2 mm) sieve and dried overnight to ensure adequate moisture removal. Next, the soil was sonicated with 1M HCl (1:3 soil/liquid ratio) at 28 kHz for 30 min and the treated soil was then analyzed following by the standard method of environmental pollutions for soil pollution [34]. The soil was then left to dry overnight, and 3 g of the dried soil sample was added to a highly corrosive/extractive mixture of strong acids (HNO_3_ 7mL + HCl 21 mL). Heavy metals were extracted from the sample using a trace metal digestion system (Gerhardt SMA20A, CM Corporation Ltd., Seoul, Korea) and then filtered using a slurry filtration system (Whatman filter paper No. 40, Whatman^®^, Pittsburgh, PA, USA). The concentrations of Pb^2+^, Cd^2+^, Zn^2+^, and Cu^2+^ in the filtered solution were analyzed with ICP-MS (ICPS-7500, Shimadzu, Kyoto, Japan) (Table S1). The ICP-MS was calibrated with an ICP multi-element standard solution IV (Merck Millipore). For a sensor performance evaluation with different temperatures, two temperatures were used at 4 °C and at 23 °C with a relative humidity of 45%. 

## 3. Results and Discussions

### 3.1. Characterization of Bi-Chitosan Modified Electrode

**Figure 2:**
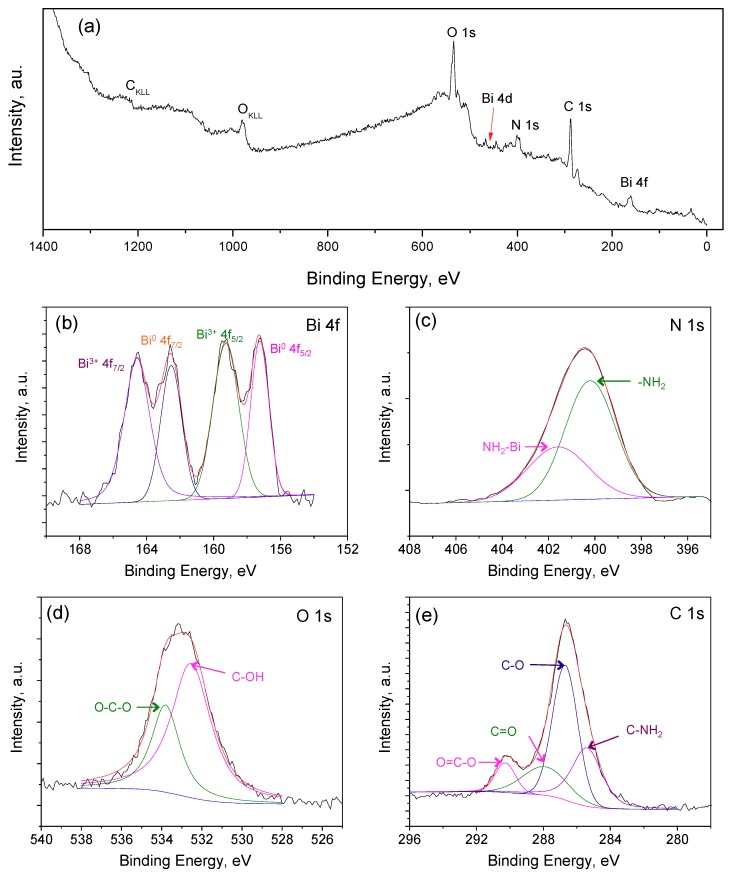
XPS analysis of a Bi-chitosan nanocomposite film. (**a**) Survey peak, (**b**) Bi 4f spectra, (**c**) N 1s spectra, (**d**) O 1s spectra, and (**e**) C 1s spectra.

The co-deposited Bi-chitosan nanocomposite film on the bare carbon electrode was characterized using XPS and SEM analysis, and a survey scan of the Bi-chitosan nanocomposite is shown in Figure 2a. The spectrum of the nanocomposite contains peaks associated with C, O, N, and Bi. The high-resolution spectrum of Bi, Bi 4f (Figure 2b) reveals four distinct peaks. The Bi 4f_5/2_ and Bi 4f_7/2_ spin orbital splitting of Bi metal are at binding energies of 157.2 eV and 162.5 eV, respectively, and these peaks and 5.3 eV of energy splitting between two orbits are in agreement with reported values elsewhere [35,36]. Similarly, spin orbital energy splitting of Bi 4f_5/2_ and Bi 4f_7/2_ is at binding energies of 159.3 eV and 164.6 eV, respectively, corresponding to a 3+ oxidation state of the Bi atom. Similar to metal Bi, the energy difference between two-spin orbital energy splitting of 3+ oxidized Bi is 5.3 eV. Therefore, it can be concluded that Bi is deposited as a mixed form of metal Bi and the 3+ oxidation state of the Bi atom. Figure 2c shows N 1s high-resolution spectrum of the composite film. The peak at 399.3 eV is assigned to the amino group involved in the hydrogen bond, whereas the peak at 401.7 eV is a result of chelation between the amino group and the Bi metal [37]. The O 1s high-resolution signal is presented in Figure 2d. The peaks observed at 532.6 and 533.8 correspond to C-OH and O-C-O, respectively [35]. Figure 2e shows C 1s spectra of the film reveals 285.4, 286.7, 287.9 and 290.1 peaks and these peaks are assigned to C-NH_2_, C-O, C=O, and O=C-O group in the metal-polymer composite film [38,39]. The XPS analysis proves that the Bi-chitosan composite material was successfully deposited on the carbon substrate.

**Figure 3:**
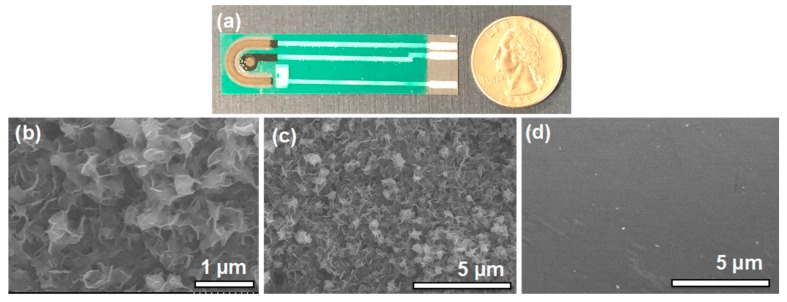
Optical image of the fabricated sensor (**a**) SEM images of a Bi-chitosan nanocomposite film at (**b**) 30 k magnification and (**c**) 10 k magnification, and (**d**) SEM image of a bare carbon electrode at 10 k magnification.

Optical images of the sensor and SEM images of the Bi-chitosan nanocomposite film on the carbon electrode are presented in Figure 3. Figure 3a shows the fabricated sensor. A uniform porous nanostructure was observed as shown in Figure 3b,c on the Bi-chitosan deposited film, providing a high surface area, desirable for improved sensitivity compared to the bare carbon electrode (Figure 3d). To investigate the effect of electrodeposition conditions on the Bi-chitosan nanocomposite film thickness and the sensitivity toward heavy metal ions, five different electrodeposition conditions (e.g., different electrodeposition times between 30 and 180 s at −100 mA/cm^2^ and different current densities (−100 vs. −200 mA/cm^2^) at 120 s of electrodeposition time) were evaluated (Figure S1). The film thickness measured from the cross-sectional image using an optical microscope was 5.2, 7.6, 9.3, and 10.1 µm with 30, 60, 120, and 180 s, respectively. With a lower current density (−200 mA/cm^2^), the thickness of the Bi-chitosan composite film was 11.5 µm for 120 s of electroplating time. (Table S2). The nanocomposite film thickness was increased with increased disposition time; as expected, time has a direct effect on film thickness. When using the same deposition time, the film thickness was increased with an increase in the current density. We have optimized the response of the sensor with respect to change in the current density and the deposition time. 

### 3.2. Electrochemical Impedance Spectroscopy of Bi-Chitosan Modified Electrode

**Figure 4:**
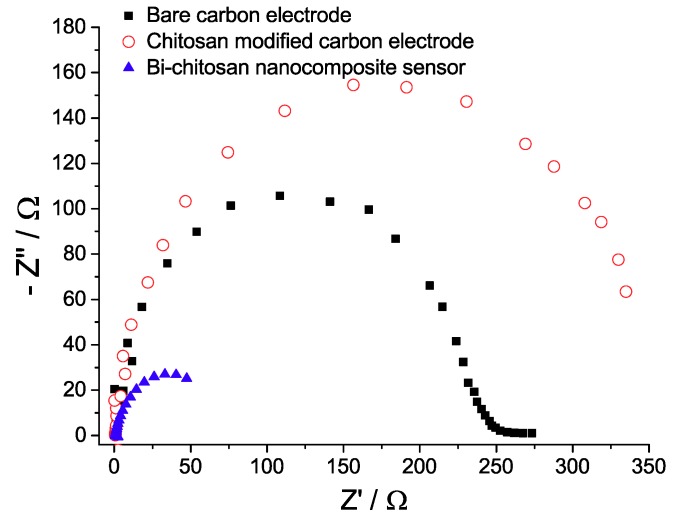
Nyquist diagrams of a bare carbon electrode, a chitosan modified carbon electrode, and a Bi-chitosan nanocomposite sensor. The impedance results were obtained using 10 mM potassium ferricyanide (K_3_(Fe(CN)_6_]) as an electrolyte solution at a frequency range 10 mHz to 500 kHz.

The electron transfer properties of the electrode after different surface modifications were determined by using EIS. It is generally known that the charge transfer resistance across the interface electrode/electrolyte is proportional to the diameter of the arc of the Nyquist plot at a constant bias potential [40]. Figure 4 shows the comparison of Nyquist plots between the bare carbon electrode, the chitosan modified carbon electrode, and the Bi-chitosan nanocomposite electrode. The Nyquist diagrams were processed using an EIS spectrum analyzer software to quantify the charge transfer resistance. The charge transfer resistance for the chitosan modified carbon electrode (398 Ω) was higher compared to the bare carbon electrode (235 Ω). This result indicates that the chitosan is hindering the charge transfer process on the fabricated electrode. In contrast, the charge transfer resistance of the Bi-chitosan modified carbon electrode (46 Ω) was 5.1–8.6 times lower compared to both previous electrodes. The value of charge transfer resistance depends on the insulating and dielectric properties at the electrode/electrolyte interface [41]. The above results illustrate that the Bi-chitosan nanocomposite film is facilitating the electron transfer process. The electrode/electrolyte interface plays a key role in the detection of analytes in electrochemical sensor. Thus, the enhanced charge transfer process of the composite material benefits the response of the sensor.

### 3.3. Optimization of SWASV Parameters for Pb(II), Cd(II) and Zn(II) Detection using the Bi-Chitosan-Coated Carbon Electrode

The effect of SWASV operational parameters (e.g., heavy metal deposition potential, deposition time, amplitude, and frequency) on heavy metal detection with the Bi-chitosan nanocomposite sensor performance were evaluated. We selected the middle value of Bi-chitosan nanocomposite thickness (7.6 µm, −100 mA/cm^2^ for 60 s) for testing operating parameters. Optimal SWASV operational conditions were determined by adjusting applied deposition potentials for heavy metals, deposition times, amplitudes and frequencies at a fixed 10 ppb Pb^2+^ concentration in 0.1 M AcB at pH 4.5 (Figure S2). At a deposition potential of −1.4 and −1.2 V similar current peaks around 43 µA were shown. A previous study reported that H_2_ gas evolution was observed in negative potentials above −1.4 V, which led to diminishing of the sensor lifetime due to dark-brown burnt materials on the working electrode along with vigorous H_2_ gas evolution [42]. Therefore, −1.2 V was selected as a suitable deposition potential that would provide the maximum Pb^2+^ stripping peak current without hydrogen gas production. The effect of deposition time on the stripping peak currents was investigated for a range of 30 to 600 s. The peak currents for Pb^2+^ increased from 8.8 to 48.6 µA with increased deposition time, due to the amplified amount of metal ions deposited on the composite film surface over time. Conversely, the peak currents were not significantly increased over time and were even deemed unstable with a deposition time longer than 300 s. A previous study reported that longer preconcentration times (e.g., 300 and 400 s) were chosen for further routine analyses [43], therefore, 300 s was deemed as the optimal and practical deposition time. As demonstrated, the amplitude and frequencies displayed significant effects on the stripping response (Figure S2). The highest current was observed at an amplitude of 0.25 V and a frequency of 20 Hz, though the peak showed an ambient noise as a result of amplitude property [44]. Therefore, the amplitude of 0.05 V was chosen as the optimal amplitude. Overall, a deposition potential of −1.2 V, deposition time of 300 s, a pulse amplitude of 0.05 V and a frequency of 20 Hz were chosen as optimal parameters and were, therefore, used to construct calibration curves for evaluating heavy metal ion detection using the sensors. These parameters are analogous to the optimal SWASV operational conditions of both Bi-coated sensors and chitosan, which are −1.2 V of deposition potential, 300 s of deposition time, 0.05 V of pulse amplitude and 20 Hz of frequency [14,45], suggesting that Bi and chitosan had no significant impact on the operational conditions for either material. 

### 3.4. Effect of Electroplating Conditions on Bi-Chitosan Nanocomposite for Detecting Heavy Metal Ions

**Figure 5:**
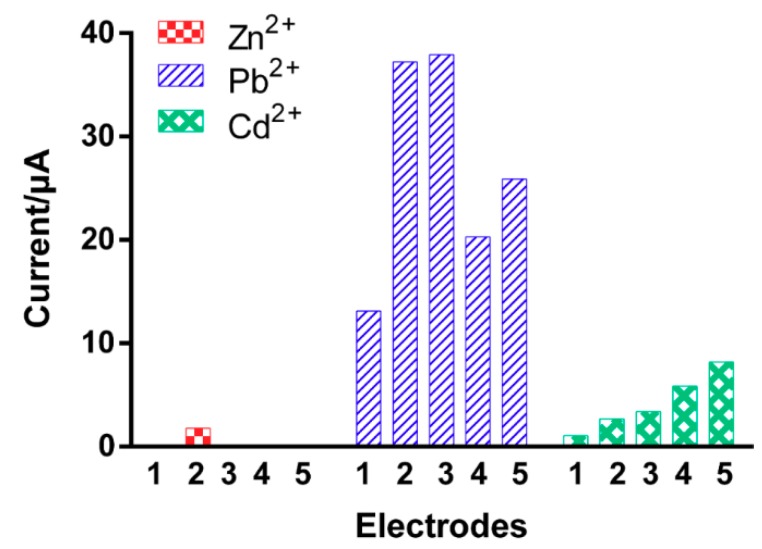
Effects of different Bi-chitosan nanocomposite preparation conditions on the detection of heavy metal ions. Electrode #1: −100 mA/cm^2^ for 30 s, Electrode #2: −100 mA/cm^2^ for 60 s, Electrode #3: −100 mA/cm^2^ for 120 s, Electrode #4: −100 mA/cm^2^ for 180s, Electrode #5: −200 mA/cm^2^ for 120 s. The heavy metal concentrations are 3.3 ppb of Zn^2+^, 5 ppb of Cd^2+^, and 10 ppb of Pb^2+^. Heavy metal deposition time is 300 s with a −1.2 V deposition potential, 0.004 V potential step, 0.05 V amplitude, and 20 Hz frequency.

Figure 5 shows the effects of different electroplating conditions on the sensitivity toward Zn^2+^, Cd^2+^, and Pb^2+^ at an equivalent mole concentration of 5 × 10^−9^ M (i.e., 3.3 ppb of Zn^2+^, 5 ppb of Cd^2+^, and 10 ppb of Pb^2+^), which were evaluated by the optimal SWASV operational conditions (300 s of deposition time, −1.2 V of deposition potential, 0.004 V of potential step, 0.05 V of amplitude, and 20 Hz of frequency). The highest current of 37.9 µA toward Pb^2+^ was obtained at -100 mA/cm^2^ for 120 s of deposition time (Electrode #3 with 9.3 µm film thickness). A similar current was also observed at a half-reduced deposition time (Electrode #2); however, 30 s was found to be relatively short for developing a sufficient Bi-chitosan nanocomposite film to deposit Pb^2+^ on the electrode. However, increasing the electroplating time (180 s) also showed a decrease in currents for detecting Pb^2+^, as low as 20.3 µA of Pb^2+^ due to a thick Bi film. The decreased stripping peak currents of Pb^2+^ that occurred for electrodes with a thicker Bi film on the surface (Electrode #4 and #5 vs. Electrode #2 and #3) is consistent with a previous study [46], suggesting that excess metal composite on the sensor surface may hinder the charge transfer of heavy metal ions during the stripping phase. Thin films can be saturated with heavy metal ions during the deposition and stripping step, while thick films have the limitation of mass transfer due to the diffusion occurring during the stripping step [47]. Therefore, the electrodes with different thicknesses show different sensing characteristics. It was found that 60-120 s of deposition time for Bi-chitosan nanocomposite was appropriate for Pb^2+^ detection. Unlike Pb^2+^, the current produced by cadmium ions (Cd^2+^) increased with increased electroplating deposition times and increased current densities (Figure 5). Electrochemical detection of Zn^2+^, however, was only observed at −100 mA/cm^2^ and 60s of deposition time (Electrode #2). From this experiment, it was concluded that for simultaneous measurements of 1.8 µA of Zn^2+^, 2.7 µA of Cd^2+^, and 37.2 µA of Pb^2+^ using the developed Bi-chitosan nanocomposite sensor, the optimal electrodeposition condition was determined at −100 mA/cm^2^ of current density and with 60 s of deposition time. With a suitable Bi thickness structure given by a deposition time of 60 s, the stability was greatly improved, being signified by an increase in sensitivity.

### 3.5. Characterization of Heavy Metal Detection in Low Concentration Solutions

**Figure 6:**
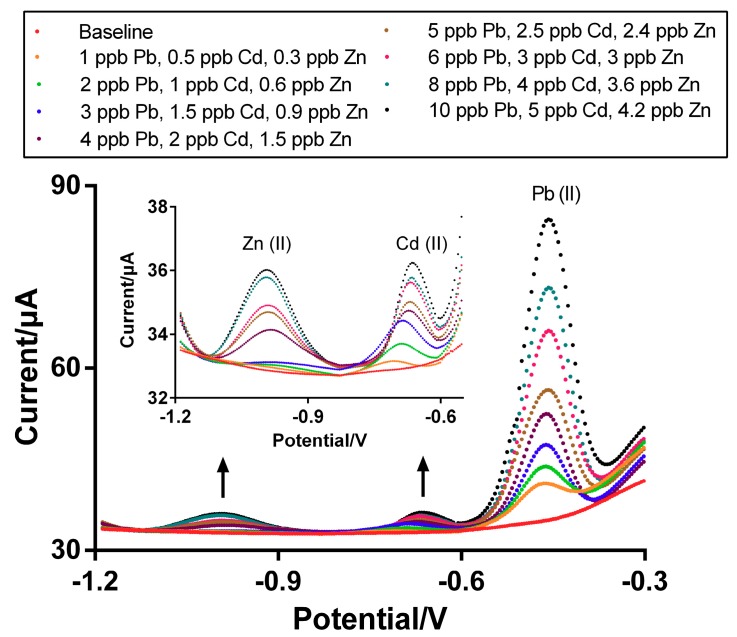
SWASV of multiple heavy metal detection (**a**) Zn^2+^, (**b**) Cd^2+^, and (**c**) Pb^2+^ in 0.1 M AcB at pH 4.5. Heavy metal deposition time is 300 s with a −1.2 V deposition potential, 0.004 V potential step, 0.05 V amplitude, and 20 Hz frequency.

Using the optimized nanocomposite film prepared at −100 mA/cm^2^ for 60 s of deposition time, simultaneous analyses of Zn^2+^, Cd^2+^, and Pb^2+^ concentrations in low ranges (1–5 ppb for Zn^2+^ and Cd^2+^ and 1–10 ppb for Pb^2+^) were conducted in a 0.1 M AcB (Figure 6) by the optimal SWASV operational conditions (300 s of deposition time, −1.2 V of deposition potential, 0.004 V of potential step, 0.05 V of amplitude, and 20 Hz of frequency). Clear peaks were observed for Zn^2+^, Cd^2+^ and Pb^2+^ at ca. −0.99, −0.63, and −0.45 V, respectively (Figure 6a–c). The corresponding values were then plotted and used to develop a calibration curve for each heavy metal. The corresponding calibration plots and correlation coefficients were I_p_ = 1.374*x* − 0.256 (R^2^ = 0.981) for Zn^2+^, I_p_ = 0.522*x* − 0.321 (R^2^ = 0.929) for Cd^2+^ I_p_ = 4.518*x* − 1.604 (R^2^ = 0.984) for Pb^2+^, (*x*: concentration (ppb) and *y*: current (μA)), respectively (Figure S3). Using the equation for a LOD calculation elsewhere [32], the LOD was determined to be 0.3 ppb of Zn^2+^, 0.4 ppb of Cd^2+^ and 1 ppb of Pb^2+^. 

The simultaneous multi heavy metal ion detection of Zn^2+^, Cd^2+^ and Pb^2+^, using the optimized Bi-chitosan nanocomposite sensor, showed improved performance compared to previous results obtained from Bi film-modified electrodes (Table 1).

The developed nanocomposite sensor in this study showed relatively lower LOD for Zn^2+^, Cd^2+^ and Pb^2+^ (less than 0.2 ppb) compared to previously reported LOD values of Zn^2+^, Cd^2+^, or Pb^2+^ using other Bi-based carbon paste electrodes [56]. In addition, compared to other Bi film-modified coated electrodes (0.003–0.044 µA/ppb of Zn^2+^ and 0.038–0.941 µA/ppb of Pb^2+^) and chitosan-coated electrodes (0.268 µA/ppb of Zn^2+^ and 0.417 µA/ppb of Pb^2+^), the developed Bi-chitosan nanocomposite sensor showed excellent sensitivity (determined as a slope in a calibration curve) for detecting heavy metal ions (1.374 µA/ppb of Zn^2+^, 0.522 µA/ppb of Cd^2+^, and 4.518 µA/ppb of Pb^2+^) (Table 1). This relatively high sensitivity of the developed Bi-chitosan nanocomposite sensor suggests that chitosan has strong adsorption towards metal ions due to the presence of amino group (-NH_2_) [57]. Furthermore, presence of hydroxide group (-OH) makes the working electrode more hydrophilic which would further enhance the sensitivity [57]. The relatively good reproducibility (*n* = 10) with 4.2% of Zn^2+^, 3.6% of Cd^2+^ and 5.1% of Pb^2+^ relative standard deviations (RSDs) along with low LOD demonstrates the excellent analytical performance of the optimized Bi-chitosan nanocomposite sensor compared previous similar sensor development. 

### 3.6. Application to a Real Wastewater Environment

**Figure 7:**
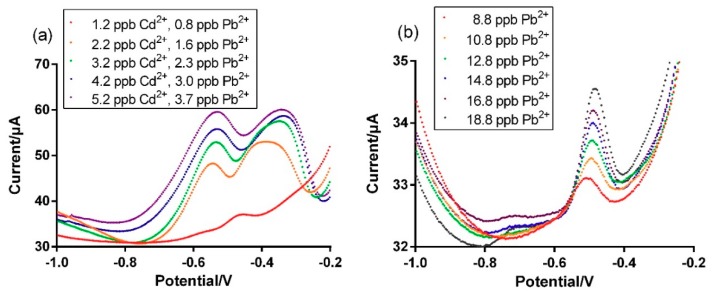
Heavy metal detection using the fabricated Bi-chitosan nanocomposite sensor in (**a**) mining wastewater and (**b**) soil leachate samples. The mining wastewater dilution factor and soil leachate dilution factor are 80 and 50,000, respectively. The spiked data for calibration curves were plotted after baseline subtraction.

The improved sensor performance by the Bi-chitosan nanocomposite for simultaneous detection of Zn^2+^, Cd^2+^, or Pb^2+^ was further evaluated using real mining wastewater containing heavy metals and soil leachates extracted from a heavy metal contaminated site. To apply an electrochemical sensor for in situ measurement in a real sample (e.g., drinking water, wastewater, and leachate), a full characterization is required to understand the electrochemical behaviors of the developed sensors toward the specific water to be tested. One of the methods for characterization involved the use of a calibration curve constructed in the same (or diluted) water by spiking it with high concentrations of the ions to be detected. In order to evaluate sensor behaviors in real samples, a calibration curve for the detection of Zn^2+^, Cd^2+^, or Pb^2+^ in each sample was constructed (Figure 7). Due to the relatively high and variable heavy metal concentrations present in the original mining wastewater and soil leachate samples (Table S1), they were diluted using DI water to obtain a relatively low concentration (Pb^2+^ was used as a reference for determining dilution factors) and spikes of the original samples were added (as a standard stock solution) to the diluted wastewater samples consecutively to construct a calibration curve for each wastewater sample. The diluted real samples showed well-defined Pb^2+^ peak currents in the range of 0 and 20 ppb (Figure 7). However, Zn^2+^ peak currents were not detected in each sample although both the original (undiluted) samples have high concentrations of Zn^2+^ (11.5 ppm for mining wastewater and 439.2 ppm for soil leachate). This may be due to an interference of the presence of Cu^2+^ in the sample (10.5 ppm for mining wastewater and 38.1 ppm for soil leachate) on Zn^2+^ detection probably due to the Cu-Zn intermetallic species formation. It is well known that Cu-Zn intermetallic species can be developed on the working electrode surface during the deposition step, interfering Cu^2+^ or Zn^2+^ detection using SWASV [58,59]. While Cd^2+^ was detected in the mining wastewater, Cd^2+^ peaks were not observed when testing the diluted soil leachate sample (Figure 7) probably due to a lower reference LOD concentration than the LOD for Cd^2+^. As the soil leachate was diluted based on Pb^2+^, with a final concentration in the range of 8 and 20 ppb, the Cd^2+^ concentration was 47 times lower that this range (Cd^2+^ ≈ 0.16 ppb). 

The sensitivity for Cd^2+^ and Pb^2+^ were 4.426 μA/ppb and 7.347 μA/ppb, respectively, for mining wastewater with a spiked concentration range of 1 to 4 ppb for Cd^2+^ and 0.7 to 2.8 ppb for Pb^2+^ (Figure S4a,b). With diluted soil leachate, the slope of the calibration curve was 0.113 μA/ppb for Pb^2+^ (Figure S4c). It should be noted that the sensitivity for Cd^2+^ and Pb^2+^ in a real sample may be different with metal ion interferences. Eight and six successive measurements with stable peak currents were recorded continuously using the Bi-chitosan nanocomposite sensor for mining wastewater and soil leachate samples, respectively. RSD and recovery were 1.3% and 106.7% for Cd^2+^ and 5.6% and 108.8% for Pb^2+^ for the mining wastewater and 3.1% of RSD and 93.4% of recovery was observed for the soil leachate sample (Table 2), indicating the excellent stability and reproducibility of the Bi-chitosan nanocomposite sensor in real wastewater. One of the advantages of using anodic stripping voltammetry (ASV) for heavy metal detection is that each heavy metal ion has its own potential window where it is anodically stripped (e.g., −1.3 to −1.1 V for Zn^2+^, −0.9 to −0.7 V for Cd^2+^, −0.6 to −0.4 for Pb^2+^, −0.2 to 0 for Cu^2+^, 0 to 0.2 for As^3+^ and 0.2 to 0.4V for Hg^2+^, respectively) [60,61,62]; thus, the presence of such metal ions theoretically shows no interference unless the potential windows are overlapped. Whereas, Zn^2+^ was not detected in real samples due to Zn^2+^ and Cu^2+^ interference possibly due to the formation of Zn-Cu intermetallic compounds (IC) during the deposition step [63]. 

### 3.7. Investigation of the Effect of Temperature on Heavy Metal Ion Detection in Real Wastewater 

**Figure 8:**
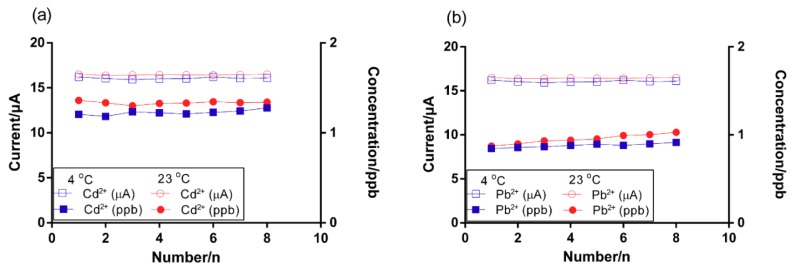

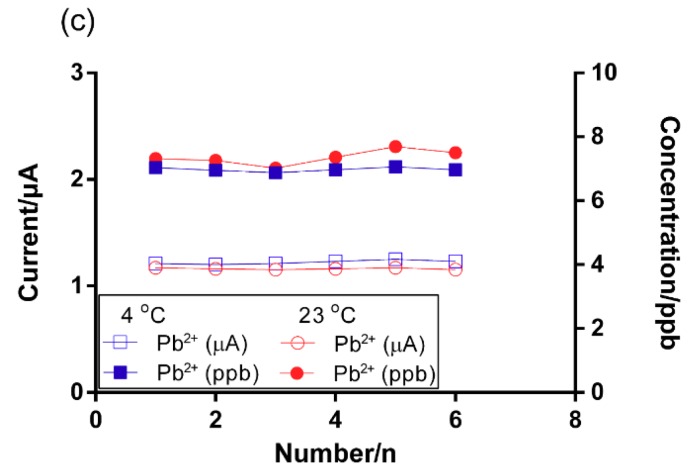
The reproducibility of heavy metal detection under different temperatures of (**a**) Cd^2+^ and (**b**) Pb^2+^ in a mining wastewater sample, and (**c**) Pb^2+^ in a soil leachate sample using the Bi/chitosan-coated carbon electrode.

To evaluate the performance of the developed Bi-chitosan nanocomposite sensor in a real wastewater environment under different temperatures such as 4 and 23 °C, experiments were conducted using mining wastewater and soil leachate. The temperature (>25 °C) can be a thermodynamically influencing parameter in electrochemical sensing in water [64]. The average current and heavy metal concentrations of mining wastewater observed were 16.4 µA and 1.3 ppb of Cd^2+^ and 16.1 µA and 1.0 ppb of Pb^2+^ at 23 °C, respectively. A similar value was observed regarding the low temperature (4 °C) for both Cd^2+^ and Pb^2+^ in mining wastewater (Figure 8a,b). There was only a decrease of 6.1% of Cd^2+^ and 4.3% of Pb^2+^ concentration in response to decreasing the temperature from 23 to 4 °C. In the soil leachate, a decrease of 4% of the highest Pb^2+^ (7.4 ppb at 23 °C vs. 7.1 ppb at 4 °C) concentration was observed (Figure 8c). These results support that low temperature had minimal impact on heavy metal detection using the Bi-chitosan nanocomposite sensor. In turn, performance results of Bi-chitosan nanocomposite sensor showed that the modification of the electrode and optimization of the operating parameters prove effective for sensitive and selective in situ determination of Cd^2+^ and Pb^2+^ in real wastewater environments, independent of temperature variation.

## 4. Conclusions

A Bi-chitosan nanocomposite sensor has been successfully designed and fabricated using a co-electrodeposition procedure. Optimized conditions provided a nanocomposite film which was successfully used for the detection of low concentrations of heavy metal ions less than 10 ppb. The Bi-chitosan nanocomposite sensor demonstrated an excellent sensitivity towards Zn^2+^, Cd^2+^ and Pb^2+^ with noticeably improved stability and reproducibility in comparison to previous results obtained from Bi modified sensors. The relatively high sensitivity of the developed Bi-chitosan nanocomposite sensor could be attributed to chitosan which has strong affinity towards metal ions due to the presence of an amino group (-NH_2_). The LOD values were 0.3 ppb for Zn^2+^, 0.4 ppb for Cd^2+^ and 1 ppb for Pb^2+^ ions and the RSDs were less than 5.1% for Zn^2+^, Cd^2+^ and Pb^2+^ during 10 repeated measurements. For real-world applications of the Bi-chitosan nanocomposite sensor, heavy metal detection in samples taken from mining wastewater and soil leachate was investigated. The RSD of the Bi-chitosan nanocomposite sensor was only 1.3% for Cd^2+^ and 5.6% for Pb^2+^ in mining wastewater and 3.1% for Pb^2+^ in soil leachate, respectively. In addition, temperature had a minimal impact on Cd^2+^ and Pb^2+^ detection for both samples, indicating that the combination of Bi and biopolymer (i.e., chitosan) can enhance the stability of the electrodes during heavy metal ion detection. The Bi-chitosan nanocomposite sensor could measure Zn^2+^ concentration in stock solutions that contained multiple heavy metal ions, but showed a limitation when applied to real wastewater samples due to chemical interferences between high concentrations of Zn^2+^ and Cu^2+^. The developed Bi-chitosan nanocomposite sensor was demonstrated for multiple heavy metal ion detection in real environmental samples obtained from mining wastewater and soil leachate. 

## Figures and Tables

**Table 1:** Comparison of heavy metal detection between the developed Bi/chitosan-coated sensor and other Bi film sensors previously reported.

Electrode Substrate	Analytical Method	LOD (ppb)			Reproducibility (*n*)	RSD (%)	Sensitivity (µA/ppb)	References
		Zn^2+^	Cd^2+^	Pb^2+^				
Bi_2_O_3_-modified SPE *	DPASV	-	2.1	1.1	8	8.4 (Cd^2+^) 7.7 (Pb^2+^)	0.027 (Cd^2+^) 0.061 (Pb^2+^)	[48]
Bi precursor compounds coated SPE	DPASV	-	0.08	0.1	8	3.1 (Cd^2+^) 2.3 (Pb^2+^)	0.019 (Cd^2+^) 0.038 (Pb^2+^)	[49]
Bi oxide SPE	SWASV	-	2.5	5	10	10 (Zn^2+^) 5 (Cd^2+^) 7 (Pb^2+^)	0.003 (Zn^2+^) 0.07 (Cd^2+^) 0.085 (Pb^2+^)	[50]
Bi nanoparticles carbon SPE	SWASV	1.3	1.7	4.9	-	2.7-7.4 (Zn^2+^, Cd^2+^, and Pb^2+^)	0.044 (Zn^2+^) 0.106 (Cd^2+^) 0.941 (Pb^2+^)	[51]
Bi nano-powder electrode	SWASV	-	0.15	0.07	5	1.3 (Cd^2+^) 4.7 (Pb^2+^)	-	[52]
Bi film SPE	SWASV	10.3	6.8	3.6	-	-	-	[53]
Bi film SPE	SWASV	0.5	0.3	0.8	-	2.7 (Zn^2+^) 3.4 (Cd^2+^) 4.5 (Pb^2+^)	0.009 (Zn^2+^) 0.203 (Cd^2+^) 0.173 (Pb^2+^)	[54]
Nano porous Bi-coated carbon SPE	SWASV	-	1.3	1.5	40	3.1 (Cd^2+^) 4.3 (Pb^2+^)	0.137 (Cd^2+^) 0.117 (Pb^2+^)	[55]
Chitosan-coated carbon SPE	SWASV	1.2	-	1	30	4.8 (Zn^2+^) 5.4 (Pb^2+^)	0.268 (Zn^2+^) 0.417 (Pb^2+^)	[45]
Bi/chitosan-coated carbon SPE	SWASV	0.1	0.1	0.2	10	4.2 (Zn^2+^) 3.6 (Cd^2+^) 5.1 (Pb^2+^)	1.374 (Zn^2+^) 0.522 (Cd^2+^) 4.518 (Pb^2+^)	This study

* SPE: screen-printed electrode.

**Table 2:** Heavy metal detection in real environmental samples (23 °C) and sensor performance validation.

Sample		Reproducibility (*n*)	RSD (%)	Analysis		Recovery ** (%)
				Bi/Chitosan-Coated Sensor	ICP-MS	
Mining wastewater	Zn^2+^	-	-	-	11.5 ppm	-
	Cd^2+^	8	1.3	106.7 ± 2.1 ppb *	100 ppb	106.7
	Pb^2+^	8	5.6	76.2 ± 6.1 ppb *	70 ppb	108.8
Soil leachate	Zn^2+^	-	-	-	439.2 ppm	-
	Cd^2+^	-	-	-	8.4 ppm	-
	Pb^2+^	6	3.1	368 ± 17 ppm *	394.4 ppm	93.4

* Heavy metal concentration = sensor detection (ppb) × dilution factor. The mining wastewater dilution factor and soil leachate dilution factor are 80 and 50,000. ** Recovery = (heavy metal ion concentration determined by the developed Bi-chitosan nanocomposite sensor/heavy metal ion concentration determined by ICP-MS) × 100%

## Supplementary Materials

The following are available online at https://www.mdpi.com/2072-666X/10/8/511/s1: Figure S1: Surface images of Bi-chitosan nanocomposite films; Figure S2: Effect of deposition potential, deposition time, amplitude, and frequency on the anodic stripping peak current of Pb^2+^ using a Bi/chitosan-coated sensor; Figure S3: Corresponding SWASV calibration curves of Zn^2+^, Cd^2+^ and Pb^2+^; Figure S4: SWASV calibration curves of Cd^2+^ and Pb^2+^ in the mining wastewater and the soil leachate; Heavy metal ion concentrations in mining wastewater and soil leachate samples is shown in Table S1; Table S2: Thickness of the Bi-chitosan nanocomposite films with different electrodeposition times and current densities.

## References

[1] Edwards M., Triantafyllidou S., Best D. (2009). Elevated blood lead in young children due to lead-contaminated drinking water. Environ. Sci. Technol..

[2] Richardson S. (2003). Disinfection by-products and other emerging contaminants in drinking water. TrAC Trends Anal. Chem..

[3] Kadirvelu K. (2001). Removal of heavy metals from industrial wastewaters by adsorption onto activated carbon prepared from an agricultural solid waste. Bioresour. Technol..

[4] Šikovec M., Novič M., Franko M. (1996). Application of thermal lens spectrometric detection to the determination of heavy metals by ion chromatography. J. Chromatogr. A.

[5] Liu S.Y., Chen Y.P., Fang F., Li S.H., Ni B.J., Liu G., Tian Y.C., Xiong Y., Yu H.Q. (2008). Innovative solid-state microelectrode for nitrite determination in a nitrifying granule. Environ. Sci. Technol..

[6] Silva S.M., Alves C.R., Muchado S.A.S., Mazo L.H., Avaca L.A. (1996). Electrochemical determination of nitrites in natural waters with ultramicroelectrodes. Electroanalysis.

[7] Nolan M.A., Kounaves S.P. (1999). Microfabricated array of iridium microdisks as a substrate for direct determination of Cu^2+^ or Hg^2+^ using square-wave anodic stripping voltammetry. Anal. Chem..

[8] Lee G.J., Lee H.M., Rhee C.K. (2007). Bismuth nano-powder electrode for trace analysis of heavy metals using anodic stripping voltammetry. Electrochem. Commun..

[9] Riso R.D., Waeles M., Pernet-Coudrier B., Le Corre P., Corre P. (2006). Determination of dissolved iron(III) in estuarine and coastal waters by adsorptive stripping chronopotentiometry (SCP). Anal. Bioanal. Chem..

[10] Tanguy V., Waeles M., Vandenhecke J., Riso R. (2010). Determination of ultra-trace Sb(III) in seawater by stripping chronopotentiometry (SCP) with a mercury film electrode in the presence of copper. Talanta.

[11] Munteanu G., Munteanu S., Wipf D.O. (2009). Rapid determination of zeptomole quantities of Pb2+ with the mercury monolayer carbon fiber electrode. J. Electroanal. Chem..

[12] Rehacek V., Hotovy I., Vojs M. (2014). Bismuth Film Voltammetric Sensor on Pyrolyzed Photoresist/Alumina Support for Determination of Heavy Metals. Electroanalysis.

[13] Yi W.J., Li Y., Ran G., Luo H.Q., Li N.B. (2012). Determination of cadmium(II) by square wave anodic stripping voltammetry using bismuth–antimony film electrode. Sens. Actuators B Chem..

[14] Wang J., Lu J., Hočevar S.B., Farias P.A.M., Ogorevc B. (2000). Bismuth-Coated Carbon Electrodes for Anodic Stripping Voltammetry. Anal. Chem..

[15] Wang J., Lu J., Kirgöz Ü.A., Hocevar S.B., Ogorevc B. (2001). Insights into the anodic stripping voltammetric behavior of bismuth film electrodes. Anal. Chim. Acta.

[16] Economou A. (2005). Bismuth-film electrodes: Recent developments and potentialities for electroanalysis. TrAC Trends Anal. Chem..

[17] Fielden P.R., Economou A. (2003). Mercury film electrodes: Developments, trends and potentialities for electroanalysis. Analyst.

[18] Hwang G.H., Han W.K., Park J.S., Kang S.G. (2008). An electrochemical sensor based on the reduction of screen-printed bismuth oxide for the determination of trace lead and cadmium. Sens. Actuators B Chem..

[19] Zanini V.I.P., Giménez R.E., Pérez O.E.L., De Mishima B.A.L., Borsarelli C.D., Pérez O.E.L. (2015). Enhancement of amperometric response to tryptophan by proton relay effect of chitosan adsorbed on glassy carbon electrode. Sens. Actuators B Chem..

[20] Luo X., Zeng J., Liu S., Zhang L. (2015). An effective and recyclable adsorbent for the removal of heavy metal ions from aqueous system: Magnetic chitosan/cellulose microspheres. Bioresour. Technol..

[21] Tran V.S., Ngo H.H., Guo W., Zhang J., Liang S., Ton-That C., Zhang X. (2015). Typical low cost biosorbents for adsorptive removal of specific organic pollutants from water. Bioresour. Technol..

[22] Vicentini F.C., Silva T.A., Pellatieri A., Janegitz B.C., Fatibello-Filho O., Faria R.C. (2014). Pb(II) determination in natural water using a carbon nanotubes paste electrode modified with crosslinked chitosan. Microchem. J..

[23] Ghalkhani M., Shahrokhian S. (2013). Adsorptive stripping differential pulse voltammetric determination of mebendazole at a graphene nanosheets and carbon nanospheres/chitosan modified glassy carbon electrode. Sens. Actuators B Chem..

[24] Kadara R.O., Jenkinson N., Banks C.E. (2009). Characterization and fabrication of disposable screen printed microelectrodes. Electrochem. Commun..

[25] Janegitz B.C., Marcolino-Junior L.H., Campana-Filho S.P., Faria R.C., Fatibello-Filho O. (2009). Anodic stripping voltammetric determination of copper(II) using a functionalized carbon nanotubes paste electrode modified with crosslinked chitosan. Sens. Actuators B Chem..

[26] Asadpour-Zeynali K., Mollarasouli F. (2014). Bismuth and Bismuth-Chitosan modified electrodes for determination of two synthetic food colorants by net analyte signal standard addition method. Cent. Eur. J. Chem..

[27] Taufik S., Yusof N.A., Tee T.W., Ramli I. (2011). Bismuth oxide nanoparticles/chitosan/modified electrode as biosensor for DNA hybridization. Int. J. Electrochem. Sci..

[28] Aragay G., Pons J., Merkoçi A. (2011). Enhanced electrochemical detection of heavy metals at heated graphite nanoparticle-based screen-printed electrodes. J. Mater. Chem..

[29] Tsukamoto T., Killion H., Miller G. (2004). Column experiments for microbiological treatment of acid mine drainage: Low-temperature, low-pH and matrix investigations. Water Res..

[30] Zhang H., Coury L.A. (1993). Effects of high-intensity ultrasound on glassy carbon electrodes. Anal. Chem..

[31] Engstrom R.C. (1982). Electrochemical pretreatment of glassy carbon electrodes. Anal. Chem..

[32] Lee W.H., Wahman D.G., Pressman J.G. (2013). Amperometric carbon fiber nitrite microsensor for in situ biofilm monitoring. Sens. Actuators B Chem..

[33] Hwang J.H., Pathak P., Wang X., Rodriguez K.L., Park J., Cho H.J., Lee W.H. (2019). A novel Fe-Chitosan-coated carbon electrode sensor for in situ As (III) detection in mining wastewater and soil leachate. Sens. Actuators B Chem..

[34] Park B., Son Y. (2017). Ultrasonic and mechanical soil washing processes for the removal of heavy metals from soils. Ultrason. Sonochem..

[35] Yin K., Cui Z.D., Yang X.J., Zhu S.L., Li Z.Y., Liang Y.Q., Zhu S. (2015). Nanocrystal Bismuth Telluride Electrocatalysts for Highly Efficient Oxygen Reduction. J. Electrochem. Soc..

[36] Moulder J.F., Stickle W.F., Sobol P.E., Bomben K.D. (1992). Handbook of X-ray Photoelectron Spectroscopy.

[37] Wang Y., Li B., Zhou Y., Jia D. (2009). In Situ Mineralization of Magnetite Nanoparticles in Chitosan Hydrogel. Nanoscale Res. Lett..

[38] López-Pérez P.M., Marques A.P., Da Silva R.M.P., Pashkuleva I., Reis R.L., Da Silva M.R.M.P. (2007). Effect of chitosan membrane surface modification via plasma induced polymerization on the adhesion of osteoblast-like cells. J. Mater. Chem..

[39] Siddhanti D.A., Nash D.J., Navarro M.A., Mills D.M., Khaniya A., Dhar B., Kaden W.E., Chumbimuni-Torres K.Y., Blair R.G. (2017). The safer and scalable mechanochemical synthesis of edge-chlorinated and fluorinated few-layer graphenes. J. Mater. Sci..

[40] Kang X., Wang J., Wu H., Aksay I.A., Liu J., Lin Y. (2009). Glucose Oxidase–graphene–chitosan modified electrode for direct electrochemistry and glucose sensing. Biosens. Bioelectron..

[41] Tang L., Feng H., Cheng J., Li J. (2010). Uniform and rich-wrinkled electrophoretic deposited graphene film: A robust electrochemical platform for TNT sensing. Chem. Commun..

[42] Jothimuthu P., Wilson R.A., Herren J., Pei X., Kang W., Daniels R., Wong H., Beyette F., Heineman W.R., Papautsky I. (2013). Zinc Detection in Serum by Anodic Stripping Voltammetry on Microfabricated Bismuth Electrodes. Electroanalysis.

[43] Fan F., Dou J., Ding A., Zhang K., Wang Y. (2013). Determination of lead by square wave anodic stripping voltammetry using an electrochemical sensor. Anal. Sci..

[44] Koper N., Leston L., Baker T.M., Curry C., Rosa P. (2016). Effects of ambient noise on detectability and localization of avian songs and tones by observers in grasslands. Ecol. Evol..

[45] Hwang J.H., Wang X., Pathak P., Rex M.M., Cho H.J., Lee W.H. (2019). Measurement. Enhanced electrochemical detection of multi-heavy metal ions using a biopolymer-coated planar carbon electrode. IEEE Trans. Instrum. Meas..

[46] Cao L., Jia J., Wang Z. (2008). Sensitive determination of Cd and Pb by differential pulse stripping voltammetry with in situ bismuth-modified zeolite doped carbon paste electrodes. Electrochim. Acta.

[47] Baldrianova L., Svancara I., Vlček M., Economou A., Sotiropoulos S. (2006). Effect of Bi(III) concentration on the stripping voltammetric response of in situ bismuth-coated carbon paste and gold electrodes. Electrochim. Acta.

[48] Lezi N., Economou A., Efstathiou C.E., Prodromidis M. (2011). A study of Bi_2_O_3_-modified screen-printed sensors for determination of Cd (II) and Pb (II) by anodic stripping voltammetry. Sens. Electroanal..

[49] Lezi N., Economou A., Dimovasilis P.A., Trikalitis P.N., Prodromidis M.I., Prodromidis M. (2012). (Mamas) Disposable screen-printed sensors modified with bismuth precursor compounds for the rapid voltammetric screening of trace Pb(II) and Cd(II). Anal. Chim. Acta.

[50] Kadara R.O., Jenkinson N., Banks C.E. (2009). Disposable Bismuth Oxide Screen Printed Electrodes for the High Throughput Screening of Heavy Metals. Electroanalysis.

[51] Rico M.Á.G., Olivares-Marín M., Gil E.P. (2009). Modification of carbon screen-printed electrodes by adsorption of chemically synthesized Bi nanoparticles for the voltammetric stripping detection of Zn (II), Cd (II) and Pb (II). Talanta.

[52] Dimovasilis P.A., Prodromidis M.I. (2016). Preparation of Screen-Printed Compatible Bismuth-Modified Sol-Gel Microspheres: Application to the Stripping Voltammetric Determination of Lead and Cadmium. Anal. Lett..

[53] Olivero J., Laprade I., Servito G. (2019). Heavy Metal Detection with Bismuth Film Electrode.

[54] Rehacek V., Hotovy I., Vojs M., Mika F. (2008). Bismuth film electrodes for heavy metals determination. Microsyst. Technol..

[55] Hwang J.-H., Wang X., Zhao D., Rex M.M., Cho H.J., Lee W.H. (2019). A novel nanoporous bismuth electrode sensor for in situ heavy metal detection. Electrochim. Acta.

[56] Švancara I., Baldrianova L., Tesařová E., Hočevar S.B., Elsuccary S.A.A., Economou A., Sotiropoulos S., Ogorevc B., Vytřas K. (2006). Recent Advances in Anodic Stripping Voltammetry with Bismuth-Modified Carbon Paste Electrodes. Electroanalysis.

[57] Saha S., Sarkar P. (2016). Differential pulse anodic stripping voltammetry for detection of As (III) by Chitosan-Fe(OH)_3_ modified glassy carbon electrode: A new approach towards speciation of arsenic. Talanta.

[58] Sanna G., Pilo M.I., Piu P.C., Tapparo A., Seeber R. (2000). Determination of heavy metals in honey by anodic stripping voltammetry at microelectrodes. Anal. Chim. Acta.

[59] Church J., Lee W.H. (2018). A novel approach for in situ monitoring of Zn2+ in citrus plants using two-step square-wave anodic stripping voltammetry. MRS Commun..

[60] Pujol L., Evrard D., Groenen-Serrano K., Freyssinier M., Ruffien-Cizsak A., Gros P. (2014). Electrochemical sensors and devices for heavy metals assay in water: The French groups’ contribution. Front. Chem..

[61] Koudelkova Z., Syrovy T., Ambrozova P., Moravec Z., Kubac L., Hynek D., Richtera L., Adam V. (2017). Determination of Zinc, Cadmium, Lead, Copper and Silver Using a Carbon Paste Electrode and a Screen Printed Electrode Modified with Chromium(III) Oxide. Sensors.

[62] Gounden D., Khene S., Nombona N. (2018). Electroanalytical detection of heavy metals using metallophthalocyanine and silica-coated iron oxide composites. Chem. Pap..

[63] Lazar B., Nishri A., Ben-Yaakov S. (1981). Mutual interferences in the determination of Zn(II) and Cu(II) in seawater by anodic stripping voltammetry. J. Electroanal. Chem. Interfacial Electrochem..

[64] Gründler P., Kirbs A., Dunsch L. (2009). Modern thermoelectrochemistry. ChemPhysChem.

