# Supplementary material for: A Novel Bismuth-Chitosan Nanocomposite Sensor for Simultaneous Detection of Pb(II), Cd(II) and Zn(II) in Wastewater
Source: Micromachines (Basel). 2019 Jul 31;10(8):511. doi: 10.3390/mi10080511 (PMC6723456; doi:10.3390/mi10080511)
Supplement: Supplementary file 1 [file micromachines-10-00511-s001.pdf]

Supplementary Material

A Novel Bismuth-Chitosan Nanocomposite Sensor for Simultaneous Detection of Pb(II), Cd(II) and Zn(II) in Wastewater

Jae-Hoon Hwang, Pawan Pathak, Xiaochen Wang, Kelsey L. Rodriguez, Hyoung J. Cho and Woo Hyoung Lee

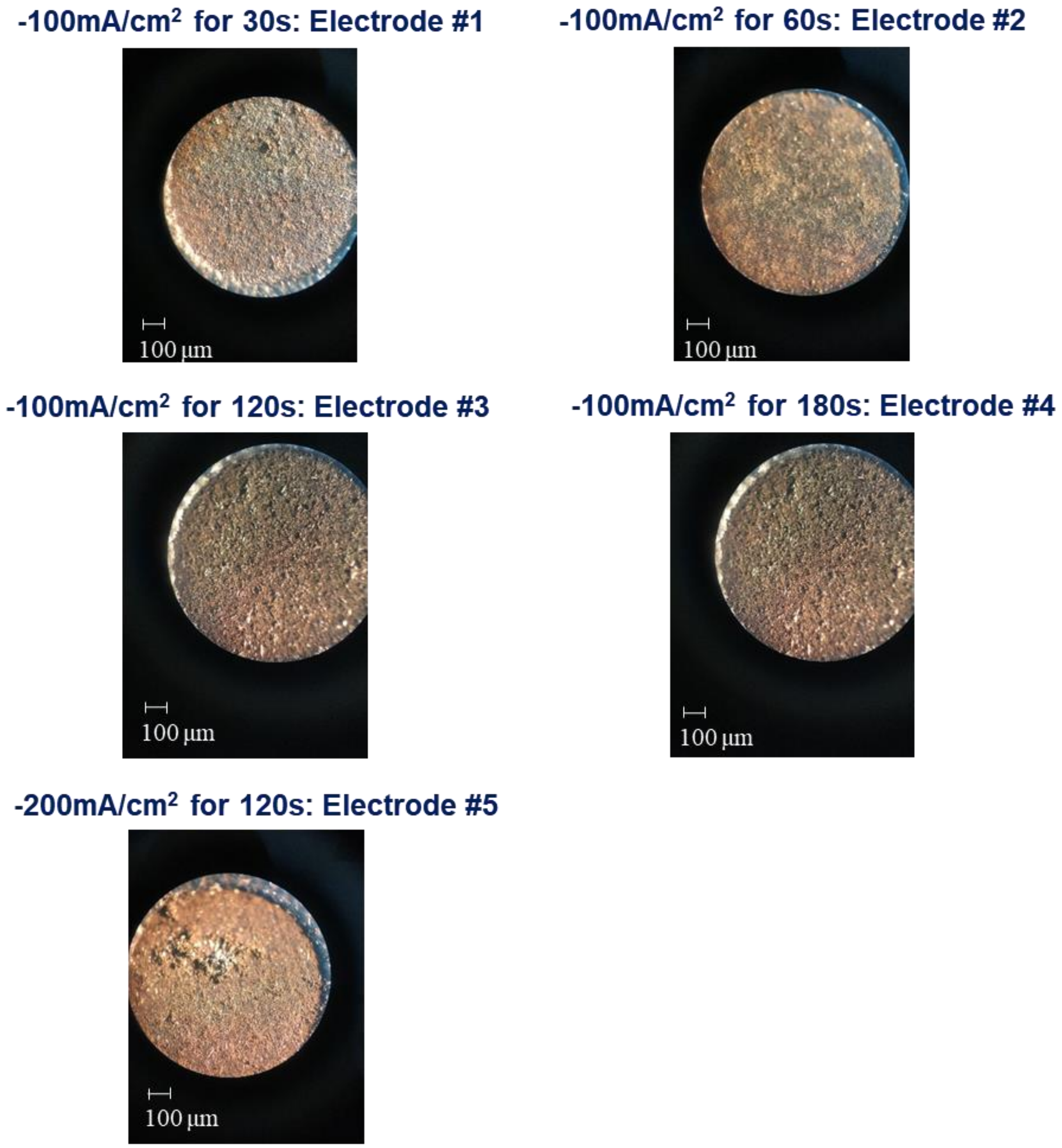

Figure S1. Surface images (×100 optical microscope) of Bi-chitosan nanocomposite films prepared under different electrochemical deposition conditions.

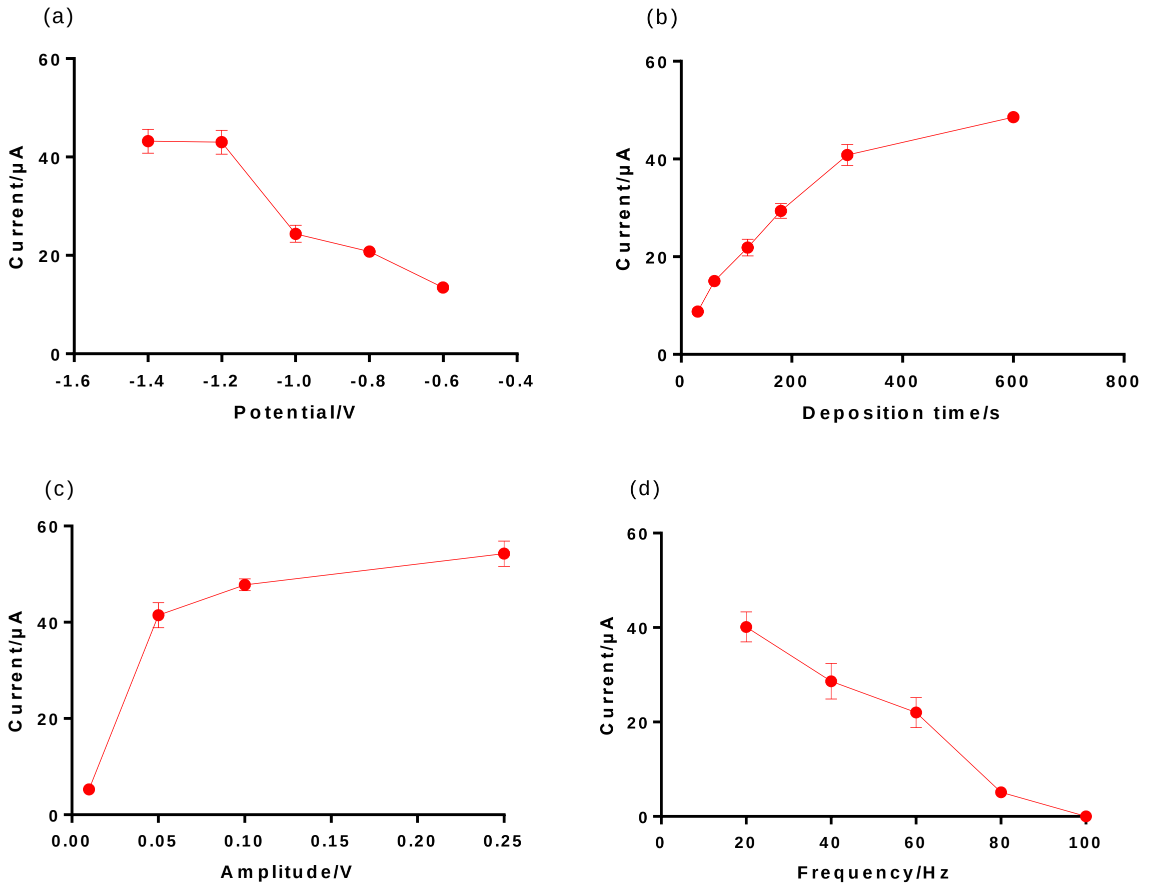

Figure S2. Effect of (**a**) deposition potential, (**b**) deposition time, (**c**) amplitude, and (**d**) frequency on the anodic stripping peak current of Pb^{2+} using a Bi/chitosan-coated sensor. Pb^{2+} concentration is 10 ppb (0.1 M AcB at pH 4.5).

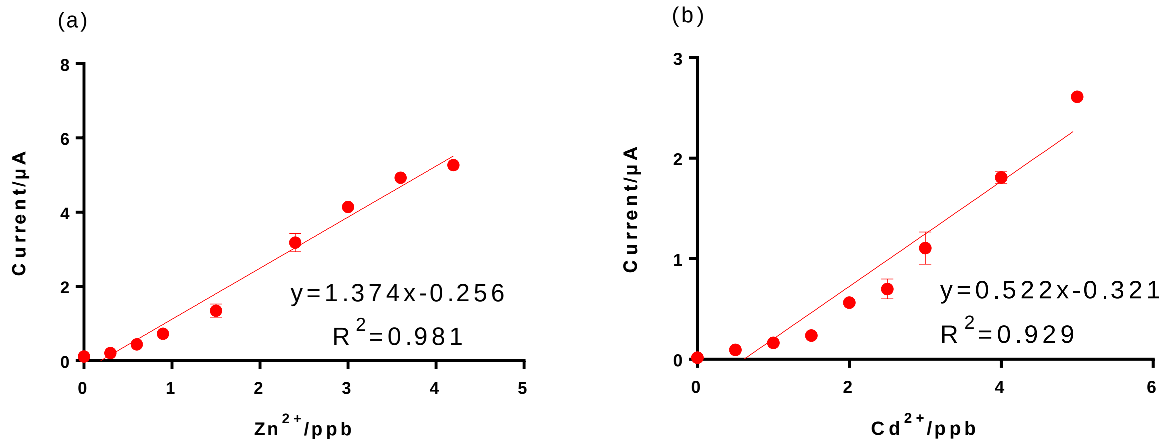

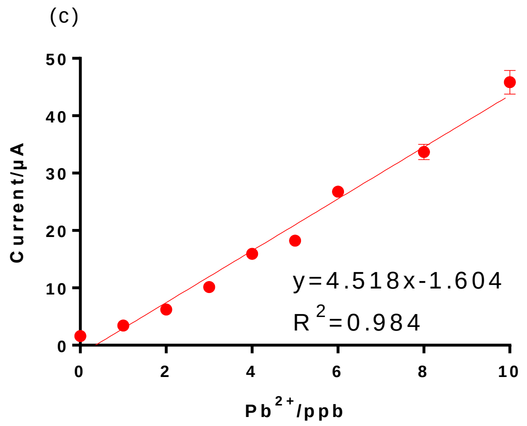

Figure S3. Corresponding SWASV calibration curves of (**a**) Zn^{2+}, (**b**) Cd^{2+} and (**c**) Pb^{2+} in 0.1 M AcB (pH 4.5). Deposition time is 300 s with a -1.2V deposition potential, 0.004 V potential step, 0.05 V amplitude, and 20 Hz frequency.

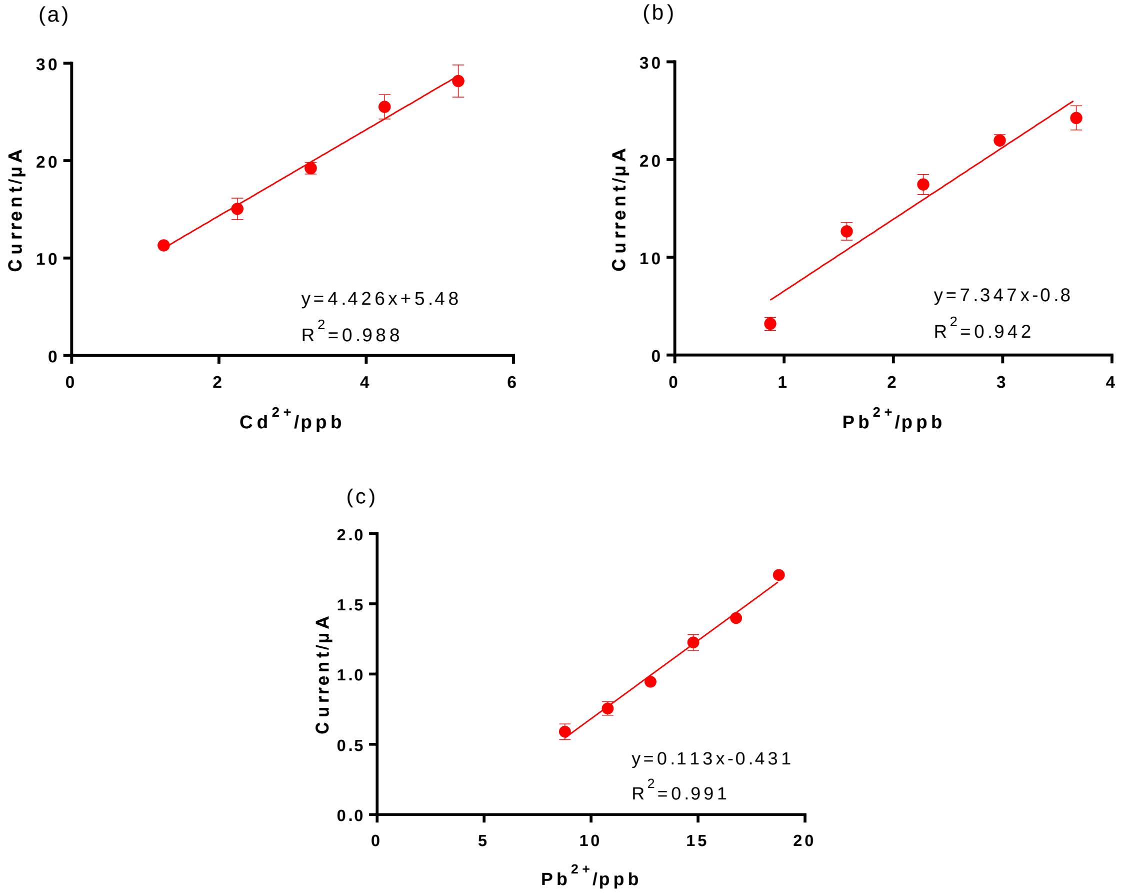

Figure S4. SWASV calibration curves of various (**a**) Cd^{2+} and (**b**) Pb^{2+} concentrations in the mining wastewater sample and (**c**) Pb^{2+} concentrations in the soil leachate.

Table S1. Heavy metal ion concentrations in mining wastewater and soil leachate samples

Sample	Heavy metal concentration (ppm)				
	Zn^{2+}	Cd^{2+}	Pb^{2+}	Cu^{2+}	As^{3+}
Mining wastewater	11.5	0.1	0.07	10.5	0.3
Soil leachate	439.2	8.4	394.4	38.1	338.5

Table S2. Thickness of the Bi-chitosan nanocomposite films with different electrodeposition times and current densities.

Supplementary Material	Supplementary Material	Supplementary Material
1	30 s, −100 mA/cm^{-2}	5.2
2	60 s, −100 mA/cm^{-2}	7.6
3	120 s, −100mA/cm^{-2}	9.3
4	180 s, −100mA/cm^{-2}	10.1
5	120 s, −200mA/cm^{-2}	11.5

© 2019 by the authors. Submitted for possible open access publication under the terms and conditions of the Creative Commons Attribution (CC BY) license (http://creativecommons.org/licenses/by/4.0/).
